# *DGAT2-MOGAT2* SNPs and Gene-Environment Interactions on Serum Lipid Profiles and the Risk of Ischemic Stroke

**DOI:** 10.3389/fcvm.2021.685970

**Published:** 2021-11-24

**Authors:** Yong-Gang Zhou, Rui-Xing Yin, Feng Huang, Jin-Zhen Wu, Wu-Xian Chen, Xiao-Li Cao

**Affiliations:** ^1^Department of Cardiology, Institute of Cardiovascular Diseases, The First Affiliated Hospital, Guangxi Medical University, Nanning, China; ^2^Department of Neurology, The First Affiliated Hospital, Guangxi Medical University, Nanning, China

**Keywords:** diacylglycerol acyltransferase 2 gene, monoacylglycerol O-acyltransferase 2 gene, single nucleotide polymorphisms, ischemic stroke, serum lipid levels, interaction

## Abstract

**Background:** The genetic susceptibility to ischemic stroke (IS) is still not well-understood. Recent genome-wide association studies (GWASes) found that several single nucleotide polymorphisms (SNPs) in the Diacylglycerol acyltransferase 2 gene (*DGAT2*) and monoacylglycerol O-acyltransferase 2 (*MOGAT2*) cluster were associated with serum lipid levels. However, the association between the *DGAT2-MOGAT2* SNPs and serum lipid phenotypes has not yet been verified in the Chinese people. Therefore, the present study was to determine the *DGAT2-MOGAT2* SNPs and gene-environment interactions on serum lipid profiles and the risk of IS.

**Methods:** Genotyping of 5 SNPs (*DGAT2* rs11236530, *DGAT2* rs3060, *MOGAT2* rs600626, *MOGAT2* rs609379, and *MOGAT2* rs10899104) in 544 IS patients and 561 healthy controls was performed by the next-generation sequencing technologies. The association between genotypes and serum lipid data was determined by analysis of covariance, and a corrected *P*-value was adopted after Bonferroni correction. Unconditional logistic regression analysis was performed to assess the association between genotypes and the risk of IS after adjustment of potential confounders.

**Results:** The rs11236530A allele was associated with increased risk of IS (CA/AA vs. CC, OR = 1.45, 95%CI = 1.12–1.88, *P* = 0.0044), whereas the rs600626G-rs609379A-rs10899104G haplotype was associated with decreased risk of IS (adjusted OR = 0.67, 95% CI = 0.48–0.93, *P* = 0.018). The rs11236530A allele carriers had lower high-density lipoprotein cholesterol (HDL-C) concentrations than the rs11236530A allele non-carriers (*P* < 0.001). The interactions of rs11236530-smoking, rs3060-smoking and rs10899104-smoking influenced serum apolipoprotein B levels, whereas the interactions of rs11236530- and rs3060-alcohol affected serum HDL-C levels (*P*_I_ < 0.004–0.001). The interaction of rs600626G-rs609379A-rs10899104G-alcohol (OR = 0.41, 95% CI = 0.22–0.76) and rs600626G-rs609379C-rs10899104T-alcohol (OR = 0.12, 95% CI = 0.04–0.36) decreased the risk of IS (*P*_I_ < 0.0001).

**Conclusions:** The rs11236530A allele was associated with decreased serum HDL-C levels in controls and increased risk of IS in patient group. The rs600626G-rs609379A-rs10899104G haplotype, the rs600626G-rs 609379A-rs10899104G-alcohol and rs600626G-rs609379C-rs10899104T-alcohol interactions were associated with decreased risk of IS. The rs11236530 SNP may be a genetic marker for IS in our study populations.

## Introduction

Blood lipid disorder is an important and modifiable risk factor for atherosclerosis (1, 2). Atherosclerosis, a progressive inflammatory disorder, is the most important cause of cardiovascular disease including ischemic stroke (IS), and its prevalence is high in middle-aged and elderly (3–5). Both dyslipidemia and IS are common chronic diseases in which multiple genetic and environmental factors and their interaction are believed to involve the risk (6–8). Evidences from family-based (9–11) and twin (12) investigations have suggested that single nucleotide polymorphisms (SNPs) could account for 10–50% of the interindividual variation in blood lipid phenotypes. A recent genome-wide association study (GWAS) showed that heritability of IS was 37.9% (13), but it varied markedly by stroke subtype: large-vessel disease, 40.3%; cardio-embolic disease, 32.6%; and small-vessel disease, 16.1%. It was also different between young-onset (42% ± 8%, *P* < 0.001) and old-onset (34% ± 10%, *P* < 0.001) stroke (14).

Previous GWASes have identified many SNPs associated with dyslipidemia (15–22) and IS (23–27) in different populations. Some SNPs involved not only in normal variation in blood lipid traits but also in extreme lipid phenotypes and impact lipid traits (28, 29). These GWASes also identified many novel SNPs associated with serum lipid parameters (15, 17–22, 28, 29). Two of these newly identified SNPs were the rs11236530 and rs499974 within the diacylglycerol acyltransferase 2 (DGAT2) and monoacylglycerol O-acyltransferase 2 (MOGAT2) genes, which were related to lower high-density lipoprotein cholesterol (HDL-C) levels in European populations (28, 29).

In the small intestine, MOGAT2 is a key enzyme responsible for triglyceride (TG) re-synthesis (30, 31). It catalyzes the synthesis of diacylglycerol from free fatty acid and monoacylglycerol, two major hydrolysis products of dietary fat (32). The MOGAT2 expression was high in human small intestine and liver (33, 34). MOGAT2 in enterocytes was the rate-limiting enzyme for the TG re-synthesis pathway (the monoacylglycerol pathway) (35). Genetic deletion of *MOGAT2* in mice altered the spatial distribution of fat absorption in the small intestine and protected against diet-induced obesity and glucose intolerance (36, 37). In a high fat diet induced mouse obese model, *MOGAT2* knockout exhibited multiple healthy metabolic phenotypes, including decreased weight, adiposity and hepatic steatosis, increased energy expenditure, and improved insulin sensitivity (31). In addition, enhanced release of anorectic gut peptides such as glucagon-like peptide-1 (GLP-1) and peptide tyrosine-tyrosine (PYY), and altered macronutrient preferences shifted away from fat were also observed in *MOGAT2* knockout mice (36, 38).

DGAT2 is also a key enzyme that catalyzes the final step of TG biosynthesis (39, 40), in which fatty acyl-CoA and diacylglycerol molecules covalently join to form TG. Overexpression of DGAT2 in mouse liver causes significant hepatic steatosis as evidenced by increased hepatic TG levels but not insulin resistance (41), whereas in obese mice induced by high-fat diet or leptin-deficiency for 7 weeks, inhibition of DGAT2 with an optimized antisense oligonucleotide resulted in marked reduction in hepatic TG as well as blood TG, diacylglycerol, and free fatty acid levels (42). *DGAT2* in humans is located at chromosome 11q13.3 comprising eight exons (43). Seventeen mutations have been identified in the coding region, the predicted promoter region, and in the 5′ non-coding exon, but the functionality of these SNPs has not been evaluated or reported (44). Moreover, most of these reported mutations are rare in the Chinese populations (45). Two of common SNPs in *DGAT2*, rs3060 and rs101988116, are located at the 3′ UTR and 5′UTR in the *DGAT2*, respectively. They have been associated with smaller liver fat changes in response to niacin treatment in patients with dyslipidemia, suggesting these non-coding polymorphisms might be related to functional effects and could affect the pharmacodynamics of niacin (45). To the best of our knowledge, however, the associations between the *DGAT2-MOGAT2* SNPs and serum lipid levels in the Chinese populations, and the susceptibility to IS have not been explored previously. Therefore, the current investigation was undertaken to detect the association between the 5 *DGAT2-MOGAT2* SNPs and serum lipid traits and IS risk in the Southern Han Chinese population.

## Methods

### Patients

This study included 544 hospitalized IS patients from our First Affiliated Hospital. There were 395 (72.6%) men and 149 (27.4%) women. The average age was 61.36 ± 13.91 years. After strict neurological examination, computed tomography, or magnetic resonance imaging (MRI), IS was diagnosed according to the Trial of Org 10,172 in Acute Stroke Treatment (TOAST) criteria (46). All of the patients with cerebral hemorrhage, cardioembolic or unspecified stroke, neoplastic or intracranial space-occupying lesion were excluded. Clinical data such as medical history, demographic characteristics and lifestyle factors were recorded on a pre-designed form and managed with Excel software. Alcohol information included questions about the number of liangs (about 50 g) or grams of rice wine, corn wine, rum, beer, or liquor consumed during the preceding 12 months. Alcohol consumption was categorized into subgroups of grams of alcohol per day: 0 (non-drinker), ≤25 and >25. Smoking status was categorized into subgroups of cigarettes per day: 0 (non-smoker), ≤20 and >20. Routine physical examination of each participant was also performed. This study was approved by the Ethics Committee of the First Affiliated Hospital, Guangxi Medical University (No. Lunshen 2014-KY-Guoji-001; Mar. 7, 2014) and was strictly conducted according to the Declaration of Helsinki. All participants signed informed consent before the investigation.

### Controls

A control group of 561 healthy subjects was also recruited from our Physical Examination Center. The age structure, sex ratio, and nationality (Han Chinese) were matched between the control and case groups. There were 413 (73.6%) males and 148 (26.4%) females. The mean age was 61.64 ± 15.49 years. All of them were free of coronary heart disease and IS at the time of medical history collection, physical examination, biochemical measurements, and imaging inspection, such as 64-slice computed tomography angiography. The subjects who took medications such as lipid-lowering agents, β adrenergic-blocking agents, thiazide diuretics, hypoglycemic agents, or hormones were excluded.

### Biochemical Assays

A fasting venous blood sample of 5 ml was obtained from each participant. A part sample of 2 ml was placed into a glass tube to perform biochemical assays, whereas another part sample of 3 ml was collected into an anticoagulant tube to extract deoxyribonucleic acid (DNA). The levels of serum HDL-C (Cholestest N HDL), low-density lipoprotein cholesterol (LDL-C, Cholestest LDL; Daiichi Pure Chemicals Co., Ltd., Tokyo, Japan), total cholesterol (TC, Tcho-1), TG (TG-LH), apolipoprotein (Apo) A1 and ApoB (RANDOX Laboratories Ltd., Ardmore, Diamond Road, Crumlin Co. Antrim, United Kingdom, BT29 4QY) in samples were determined using an autoanalyzer (Type 7170A; Hitachi Ltd., Tokyo, Japan) in our Clinical Science Experiment Center (47).

### SNP Selection and Genotyping

SNP selection was according to the following conditions: (1) SNPs were established by Haploview (Broad Institute of MIT and Harvard, USA, version 4.2); (2) SNP information was obtained from NCBI dbSNP Build 132 (http://www.Ncbi.nlm.nih.gov/SNP/); (3) The minor allele frequency (MAF) of the SNPs was higher than 5%; and (4) SNPs might be associated with blood lipid levels or atherosclerotic cardiovascular disease in recent research reports (28, 29).

Genomic DNA was extracted from peripheral blood leukocytes using the phenol-chloroform method. Genotyping of the 5 *DGAT2-MOGAT2* SNPs was performed on the Snapshot of next generation sequencing technology platform HiSeq XTen (Illumina, USA) in Sangon Biotech Co., Ltd. (Shanghai, China) (48). The forward and backward primers for *DGAT2* rs11236530, *DGAT2* rs3060, *MOGAT2* rs600626, *MOGAT2* rs609379, and *MOGAT2* rs10899104 SNPs were 5′-ACCTTTGACTACTCATTCCAACTTCTT-3′ and 5′-CCACAGGTCTGTGTTACAAGAAGA-3′; 5′-GTCATTATCTGGAGTACTAAGGTGCATAA-3′ and 5′-GGCATCATAGACAACCTGAGCAAA-3′; 5′-TCTATGGCTATGCTTCTGCAAGAA-3′ and 5′-CCCAAAGAACCAGATGTCATCGTTT-3′; 5′-AGTGTTTCCATGCATTGAGCTAGAT-3′ and 5′-GGACATGGCCTCTGCAATTTATTTATTTA-3′; and 5′-CTACATGAAGCAACTCAGCTTATCCTAAC-3′ and 5′-GATAGTAACTCCTGTCTTGAAGGTATGG-3′; respectively.

### Diagnostic Criteria

In our Clinical Science Experiment Center, the normal reference values of serum lipid parameters were TC (3.10–5.17 mmol/L), TG (0.56–1.70 mmol/L), HDL-C (0.91–1.81 mmol/L), LDL-C (2.70–3.20 mmol/L), ApoA1 (1.00–1.78 g/L), ApoB (0.63–1.14 g/L), and ApoA1/ApoB ratio (1.00–2.50) (47, 48). The diagnostic criteria of hyperlipidemia, type 2 diabetes, hypertension, overweight, and obesity are as follows: (1) Hyperlipidemia: TC > 5.17 mmol/L, and/or TG > 1.70 mmol/L (47, 48); (2) Type 2 diabetes: fasting glucose (FPG) ≥ 7.0 mmol/L, 2 h postprandial glucose ≥ 11.1 mmol/L, or self-reported diagnosis of diabetes or use of anti-diabetic medications (49); (3) Hypertension: systolic blood pressure (SBP) ≥ 140 mmHg and/or diastolic blood pressure (DBP) ≥ 90 mmHg, or self-reported diagnosis of hypertension or use of antihypertensive drugs (50); (4) Overweight: body mass index (BMI) 24–28 kg/m^2^; and (5) Obesity: BMI > 28 kg/m^2^ (47, 48).

### Statistical Analysis

Statistical software package SPSS 21.0 (SPSS Inc., Chicago, Illinois) was used to finish the statistical analyses. Pair-wise linkage disequilibrium (LD) among the SNPs was expressed by *D'* and *r*^2^ which were determined using the SHEsis software (51). The frequency of haplotypes was analyzed by means of the algorithms implemented in the PHASE program. Normally distributed quantitative data and qualitative parameters were expressed as mean ± SD (Non-normally distributed TG levels, medians and interquartile ranges) and percentages, respectively. Allele frequency was determined via direct counting, and the Hardy-Weinberg equilibrium (HWE) was estimated by the standard goodness-of-fit test. The differences in qualitative variables between the groups were evaluated by the chi-square analyses, whereas the clinical characteristics were tested by the Student's unpaired *t*-test. Analysis of covariance (ANCOVA) was used to assess the association between genotypes and serum lipid parameters, and a corrected *P*-value was adopted after Bonferroni correction. Several confounding factors including sex, age, BMI, blood pressure, alcohol consumption, and cigarette smoking were adjusted for the statistical analyses. Unconditional logistic regression analysis was performed to assess the association between genotypes and the IS risk, and to calculate odds ratio (OR) and 95% confidence interval (95% CI) after adjusting for potential confounders. The SNP-SNP and haplotype-environment interactions on serum lipid levels and the risk of IS were determined by the factorial regression analyses after controlling for potential confounders.

## Results

### Clinical Characteristics and Serum Lipid Levels

The clinical characteristics of the participants are summarized in Table 1. There was not significantly different in sex ratio, age structure, the percentages of subjects who smoked cigarettes, and the levels of LDL-C and ApoB between the two groups. The average values of SBP, DBP, pulse pressure, BMI, and TG were higher in IS than in control groups (*P* < 0.001 for all), whereas the percentages of subjects who consumed alcohol, the ApoA1/ApoB ratio, and the mean levels of TC, HDL-C and ApoA1 were lower in IS patients than in controls (*P* < 0.01 for all).

**Table 1:** Clinical characteristics and serum lipid concentrations between control and IS groups.

**Parameter**	**Control**	**IS**	***P***
Number	561	544	–
Male/Female	413/148	395/149	0.705
Age (years)	61.64 ± 15.49	61.36 ± 13.91	0.748
Body mass index (kg/m^2^)	22.57 ± 3.13	23.94 ± 3.87	<0.001
Systolic blood pressure (mmHg)	130.00 ± 19.96	148.20 ± 23.10	<0.001
Diastolic blood pressure (mmHg)	81.75 ± 11.70	84.57 ± 11.76	<0.001
Pulse pressure (mmHg)	48.25 ± 15.49	63.63 ± 17.49	<0.001
**Cigarette smoking**, ***n*** **(%)**			
Non-smoker	341 (60.78)	326 (59.93)	
≤ 20 cigarettes/day	158 (28.16)	144 (26.47)	
>20 cigarettes/day	62 (11.05)	74 (13.60)	0.410
**Alcohol consumption**, ***n*** **(%)**			
Non-drinker	326 (58.11)	365 (67.10)	
≤ 25 g/day	174 (31.02)	135 (24.82)	
>25 g/day	61 (10.87)	44(8.09)	0.008
Total cholesterol (mmol/L)	4.90 ± 1.04	4.55 ± 1.03	<0.001
Triglyceride (mmol/L)	1.01 (0.82)	1.39 (0.93)	<0.001
High-density lipoprotein cholesterol (mmol/L)	1.89 ± 0.43	1.24 ± 0.38	<0.001
Low-density lipoprotein cholesterol (mmol/L)	2.71 ± 0.83	2.69 ± 0.85	0.770
Apolipoprotein (Apo) A1 (g/L)	1.41 ± 0.26	1.03 ± 0.24	<0.001
ApoB (g/L)	0.92 ± 0.19	0.91 ± 0.20	0.553
ApoA1/ApoB	1.60 ± 0.45	1.19 ± 0.40	<0.001

*IS, ischemic stroke. Non-normally distributed triglyceride concentrations were presented as median (interquartile range), and the differences were determined by the Wilcoxon-Mann-Whitney test*.

### Genotypic and Allelic Frequencies

The genotypic and allelic frequencies of the 5 SNPs are presented in Table 2. All SNPs exhibited the HWE in both patient and control groups (*P* > 0.05 for all). The genotypic and allelic frequencies of the rs11236530 SNP was different between IS patients and controls (*P* ≤ 0.001 for each), the rs11236530A allele frequency was higher in IS than in control groups (27.85 vs. 21.12%, *P* < 0.001). There were no significant differences in genotypic and allelic frequencies of the remaining four SNPs.

**Table 2:** Genotype and allele frequencies of 5 *DGAT2-MOGAT2* SNPs in control and IS groups [*n* (%)].

**Genotype/Allele**	**Control**	**IS**	**χ^2^**	***P***
***DGAT2*** **rs11236530**				
CC	346 (61.68)	284 (52.21)		
CA	193 (34.40)	217 (39.89)		
AA	22 (3.92)	43 (7.90)	14.033	0.001
*P*_HWE_	0.442	0.863		
C	885 (78.88)	785 (72.15)		
A	237 (21.12)	303 (27.85)	13.535	<0.001
***DGAT2*** **rs3060**				
CC	29 (5.17)	46 (8.46)		
CT	216 (38.50)	210 (38.60)		
TT	316 (56.33)	288 (52.94)	4.975	0.083
*P*_HWE_	0.308	0.382		
C	274 (24.42)	302 (27.76)		
T	848 (75.58)	786 (72.24)	3.191	0.074
***MOGAT2*** **rs600626**				
AA	332 (59.18)	293 (53.86)		
AG	197 (35.12)	220 (40.44)		
GG	32 (5.70)	31 (5.70)	3.457	0.178
*P*_HWE_	0.698	0.216		
A	861 (76.74)	806 (74.08)		
G	261 (23.26)	282 (25.92)	2.104	0.147
***MOGAT2*** **rs609379**				
AA	17 (3.03)	26 (4.78)		
CA	158 (28.16)	164 (30.15)		
CC	386 (68.81)	354 (65.07)	3.119	0.210
*P*_HWE_	0.865	0.219		
A	192 (17.11)	216 (19.85)		
C	930 (82.89)	872 (80.15)	2.756	0.097
***MOGAT2*** **rs10899104**				
TT	329 (58.65)	295 (54.23)		
TG	204 (36.36)	210 (38.60)		
GG	28 (4.99)	39 (7.17)	3.485	0.175
*P*_HWE_	0.614	0.846		
T	862 (76.83)	800 (73.53)		
G	260 (23.17)	288 (26.47)	3.221	0.073

*IS, ischemic stroke; HWE, Hardy-Weinberg equilibrium*.

### Genotypes and the Risk of IS

As shown in Table 3, the rs11236530 SNP was associated with increased risk of IS (OR = 1.45, 95% CI = 1.12–1.88, *P* = 0.0044 for dominant model: CA/AA vs. CC; and OR = 1.41, 95% CI = 1.14–1.74, *P* = 0.0015 for log-additive model: A vs. C). But no association was found in the remaining 4 SNPs.

**Table 3:** *DGAT2-MOGAT2* SNPs and the risk of IS.

**SNP/model**	**Ref. genotype**	**Effect genotype**	**OR (95% CI)**	***P***
***DGAT2*** **rs11236530**				
Codominant	CC	CA	1.38 (1.05–1.80)	0.0063
		AA	2.10 (1.18–3.74)	
Dominant	CC	CA-AA	1.45 (1.12–1.88)	0.0044
Recessive	CC-CA	AA	1.85 (1.05–3.27)	0.031
Overdominant	CC-AA	CA	1.29 (0.99–1.68)	0.06
Log-additive	–	–	1.41 (1.14–1.74)	0.0015
***DGAT2*** **rs3060**				
Codominant	TT	CT	1.11 (0.85–1.45)	0.21
		CC	1.58 (0.93–2.67)	
Dominant	TT	CT-CC	1.17 (0.91–1.51)	0.23
Recessive	TT-CT	CC	1.52 (0.91–2.54)	0.11
Overdominant	TT-CC	CT	1.06 (0.81–1.37)	0.68
Log-additive	–	–	1.18 (0.96–1.45)	0.11
***MOGAT2*** **rs600626**				
Codominant	AA	AG	1.18 (0.91–1.55)	0.39
		GG	1.27 (0.72–2.23)	
Dominant	AA	AG-GG	1.19 (0.92–1.55)	0.18
Recessive	AA-AG	GG	1.18 (0.68–2.06)	0.55
Overdominant	AA-GG	AG	1.16 (0.94–1.43)	0.28
Log-additive	–	–	1.16 (0.94–1.43)	0.18
***MOGAT2*** **rs609379**				
Codominant	CC	CA	1.03 (0.78–1.37)	0.61
		AA	1.39 (0.72–2.70)	
Dominant	CC	CA-AA	1.07 (0.82–1.40)	0.62
Recessive	CC-CA	AA	1.38 (0.72–2.66)	0.33
Overdominant	CC-AA	CA	1.01 (0.77–1.34)	0.92
Log-additive	–	–	1.09 (0.87–1.37)	0.45
***MOGAT2*** **rs10899104**				
Codominant	TT	TG	1.16 (0.89–1.51)	0.54
		GG	1.12 (0.65–1.94)	
Dominant	TT	TG-GG	1.15 (0.89–1.49)	0.27
Recessive	TT-TG	GG	1.06 (0.62–1.81)	0.84
Overdominant	TT-GG	TG	1.15 (0.88–1.49)	0.30
Log-additive	–	–	1.11 (0.90–1.37)	0.33

*IS, ischemic stroke; OR, odds ratio; CI, confidence interval*.

### Haplotype Frequencies and the Risk of IS

**Figure 1:**
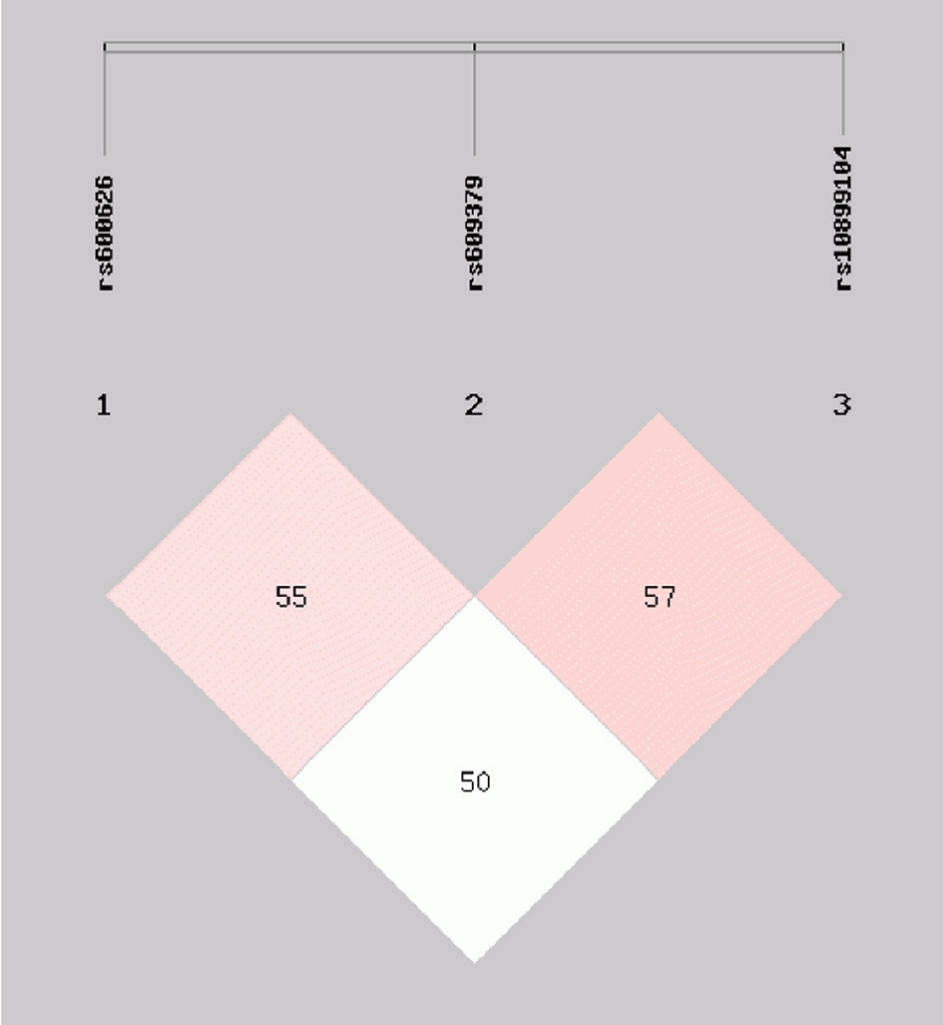
Linkage disequilibrium (LD) analysis within *MOGAT2* region (SNPs in LD) in control and IS groups. *D'* > 0.5.

Moderate LD among the rs600626, rs609379 and rs10899104 SNPs within *MOGAT2* region (SNPs in LD) was noted in controls and patients (*D'* > 0.5; Figure 1). Haplotype analyses were carried out among the 3 SNPs. Four major haplotypes are listed in Table 4. The commonest haplotype is rs600626A-rs609379C-rs10899104T. The rs600626G-rs609379A-rs10899104G haplotype was associated with decreased risk of IS (adjusted OR = 0.67, 95% CI = 0.48–0.93, *P* = 0.018).

**Table 4:** Haplotype frequencies among the *MOGAT2* rs600626, rs609379 and rs10899104 SNPs and the risk of IS.

**Haplotype**	**Total freq**.	**Control freq**.	**IS freq**.	**OR (95%CI)**	***P***
A-C-T	0.6123	0.6519	0.6146	1.00	
G-A-G	0.1143	0.1342	0.1140	0.67 (0.48–0.93)	0.018
G-C-T	0.0799	0.0859	0.0777	1.00 (0.72–1.39)	0.99
A-C-G	0.0799	0.0786	0.0777	0.83 (0.59–1.16)	0.27

*IS, ischemic stroke; OR, odds ratio; CI, confidence interval; freq., frequency*.

### Genotypes and Serum Lipid Levels in Controls

As described in Table 5, the average serum HDL-C concentrations were different among the rs11236530 genotypes (*P* < 0.001; *P* < 0.001 was considered statistical significance after the Bonferroni correction, 5 SNPs × 7 serum lipid phenotypes). Serum HDL-C concentrations in the rs11236530A allele carriers were lower than those in the rs11236530A allele non-carriers.

**Table 5:** Genotypes of the 5 *DGAT2-MOGAT2* SNPs and serum lipid levels in controls.

**SNP/** **genotype**	***n***	**TC** **(mmol/L)**	**TG** **(mmol/L)**	**HDL-C** **(mmol/L)**	**LDL-C** **(mmol/L)**	**ApoA1** **(g/L)**	**ApoB** **(g/L)**	**ApoA1/** **ApoB**
***DGAT2*** **rs11236530**								
CC	346	4.82 ± 1.09	1.00 (0.78)	1.96 ± 0.45	2.65 ± 0.84	1.41 ± 0.28	0.89 ± 0.20	1.65 ± 0.46
CA	193	5.06 ± 0.95	1.06 (0.94)	1.83 ± 0.37	2.79 ± 0.82	1.42 ± 0.22	0.92 ± 0.19	1.61 ± 0.42
AA	22	4.79 ± 0.87	0.94 (0.75)	1.74 ± 0.41	2.87 ± 0.65	1.36 ± 0.29	0.89 ± 0.13	1.63 ± 0.42
*F*		3.932	2.541	7.852	2.734	0.937	2.337	0.599
*P*		0.020	0.281	<0.001	0.066	0.393	0.098	0.550
***DGAT2*** **rs3060**								
TT	316	4.82 ± 1.10	0.98 (0.76)	1.94 ± 0.44	2.67 ± 0.84	1.40 ± 0.28	0.89 ± 0.20	1.64 ± 0.46
CT	216	5.04 ± 0.96	1.07 (0.93)	1.85 ± 0.39	2.78 ± 0.82	1.42 ± 0.22	0.92 ± 0.19	1.61 ± 0.42
CC	29	4.79 ± 0.93	1.23 (0.89)	1.93 ± 0.49	2.53 ± 0.63	1.44 ± 0.27	0.84 ± 0.13	1.76 ± 0.45
*F*		3.546	1.851	3.314	1.812	0.693	2.144	0.993
*P*		0.029	0.396	0.067	0.164	0.501	0.118	0.371
***MOGAT2*** **rs600626**								
AA	332	4.83 ± 1.16	1.01 (0.77)	1.94 ± 0.46	2.70 ± 0.88	1.41 ± 0.28	0.90 ± 0.21	1.62 ± 0.45
AG	197	5.01 ± 0.79	1.00 (0.96)	1.87 ± 0.37	2.73 ± 0.72	1.43 ± 0.21	0.90 ± 0.17	1.65 ± 0.42
GG	32	4.91 ± 1.03	1.10 (0.57)	1.86 ± 0.35	2.62 ± 0.87	1.32 ± 0.21	0.86 ± 0.21	1.64 ± 0.59
*F*		2.322	0.043	1.608	0.473	2.009	1.077	0.341
*P*		0.098	0.979	0.201	0.632	0.135	0.341	0.711
***MOGAT2*** **rs609379**								
CC	386	4.90 ± 1.11	1.05 (0.71)	1.94 ± 0.44	2.70 ± 0.86	1.41 ± 0.27	0.90 ± 0.20	1.63 ± 0.45
CA	158	4.93 ± 0.90	0.97 (0.89)	1.85 ± 0.37	2.78 ± 0.76	1.42 ± 0.22	0.90 ± 0.17	1.64 ± 0.43
AA	17	4.54 ± 0.58	1.18 (1.05)	1.80 ± 0.44	2.23 ± 0.38	1.32 ± 0.22	0.91 ± 0.26	1.54 ± 0.49
*F*		1.055	2.081	3.600	4.038	1.331	0.451	0.782
*P*		0.349	0.353	0.030	0.018	0.265	0.637	0.458
***MOGAT2*** **rs10899104**								
TT	329	4.88 ± 1.14	1.01 (0.80)	1.93 ± 0.45	2.71 ± 0.87	1.40 ± 0.29	0.90 ± 0.21	1.63 ± 0.46
TG	204	4.95 ± 0.86	1.00 (0.81)	1.88 ± 0.39	2.72 ± 0.77	1.41 ± 0.22	0.89 ± 0.18	1.65 ± 0.43
GG	28	4.76 ± 1.04	1.05 (0.81)	1.91 ± 0.41	2.58 ± 0.70	1.44 ± 0.22	0.95 ± 0.17	1.56 ± 0.36
*F*		1.062	0.121	1.206	0.946	0.327	0.401	0.209
*P*		0.346	0.941	0.300	0.389	0.722	0.670	0.811

*SNP, TC, TG, HDL-C, LDL-C, ApoA1 and ApoB are the abbreviations of single nucleotide polymorphism, total cholesterol, triglyceride, high-density lipoprotein cholesterol, low-density lipoprotein cholesterol, apolipoprotein A1 and apolipoprotein B; respectively. P < 0.001 was considered statistical significance after the Bonferroni correction (5 SNPs × 7 serum lipid phenotypes)*.

### SNP-Smoking/Drinking Interactions on Serum Lipid Levels

**Figure 2:**
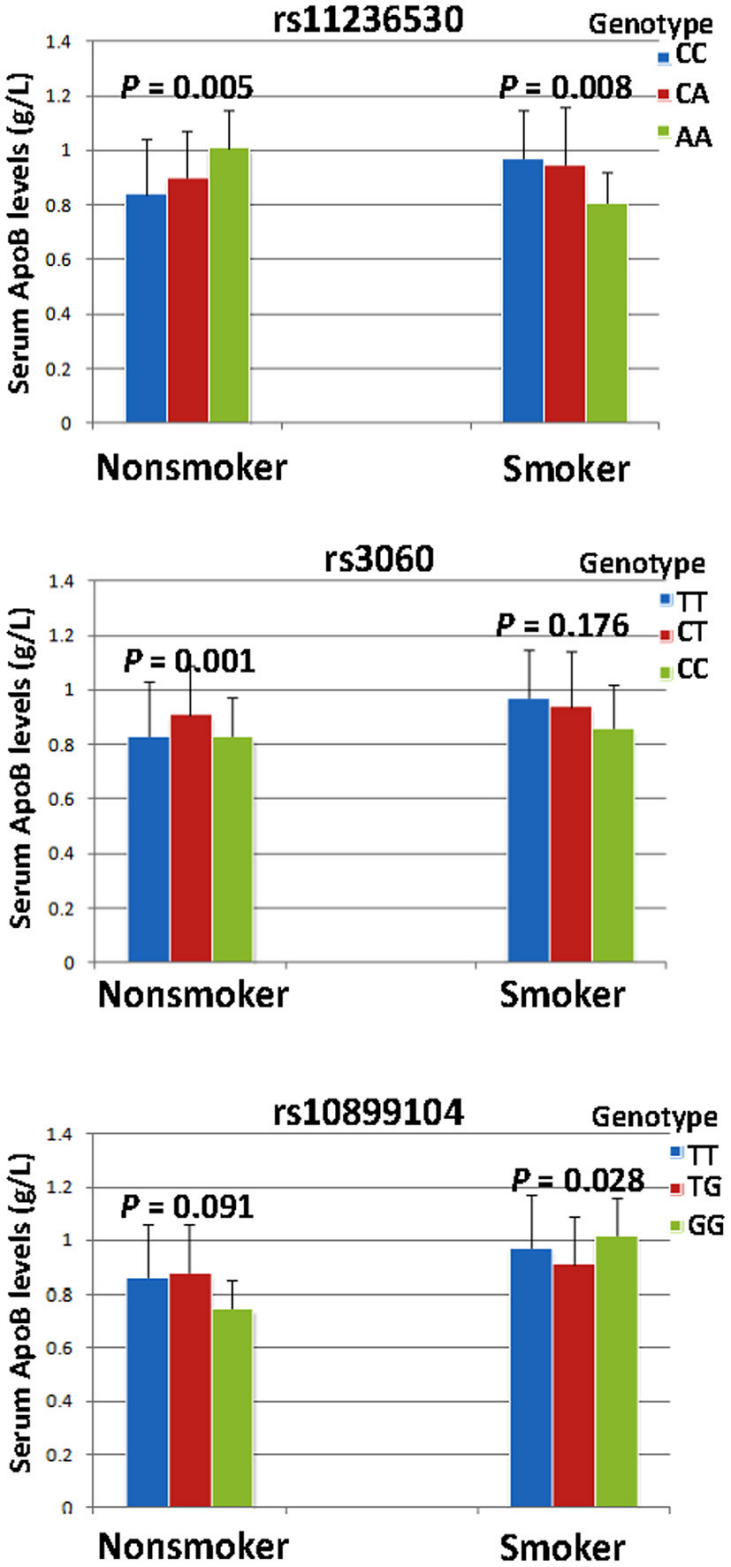
The rs11236530-smoking, rs3060-smoking and rs10899104-smoking interactions on serum ApoB concentrations. *P*_I_ ≤ 0.004; *P*_I_ < 0.005 was considered statistical significance after the Bonferroni correction (5 SNPs × 2 risk factors).

**Figure 3:**
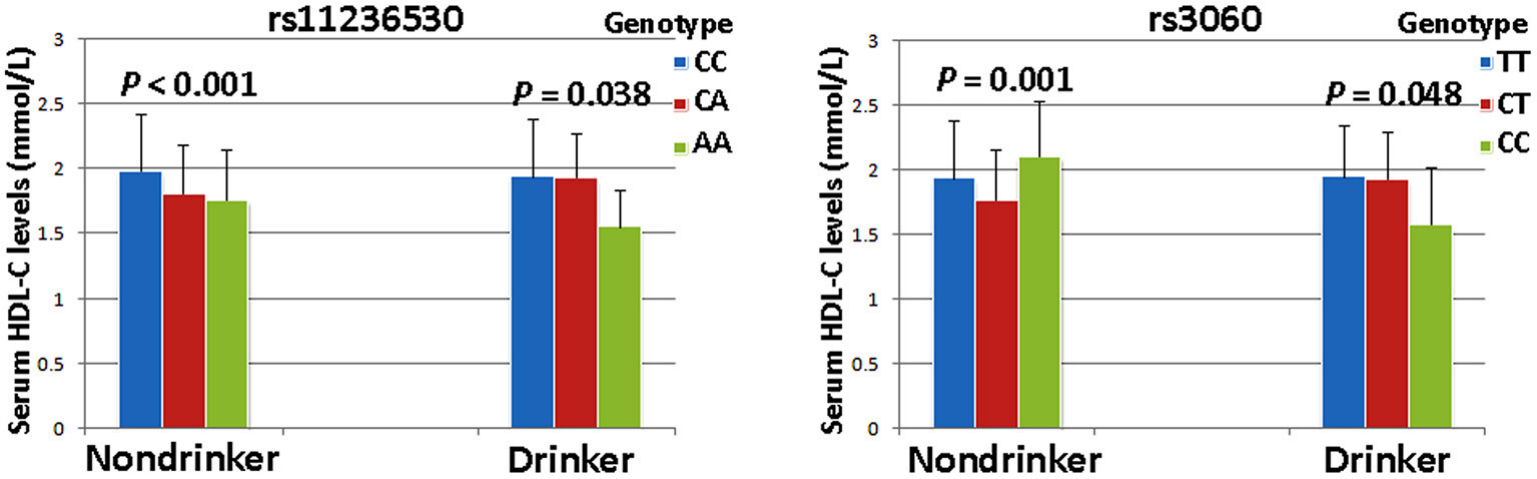
The rs11236530-alcohol and rs3060-alcohol interactions on serum HDL-C concentrations. *P*_I_ ≤ 0.004; *P*_I_ < 0.005 was considered statistical significance after the Bonferroni correction (5 SNPs × 2 risk factors).

The interaction *P-*value (*P*_I_) of SNP-smoking/drinking on serum lipid concentrations in control group is listed in Table 6. The rs11236530, rs3060 and rs10899104 SNPs interacted with **s**moking to influence serum ApoB levels (*P*_I_ < 0.004–0.001; *P*_I_ < 0.005 was considered statistical significance after the Bonferroni correction: 5 SNPs × 2 risk factors; Figure 2). The rs11236530 and rs3060 SNPs interacted with alcohol to affect serum HDL-C concentrations (*P*_I_ < 0.004–0.001; Figure 3).

**Table 6:** The interaction *P-*value (*P*_I_) of SNP-smoking/drinking on serum lipid levels in controls.

**SNP/** **factor**	**TC** **(mmol/L)**	**TG** **(mmol/L)**	**HDL-C** **(mmol/L)**	**LDL-C** **(mmol/L)**	**ApoA1** **(g/L)**	**ApoB** **(g/L)**	**ApoA1/** **ApoB**
***DGAT2*** **rs11236530**							
Smoking	0.011	0.227	0.461	0.038	0.068	0.001	0.395
Drinking	0.157	0.757	0.004	0.121	0.125	0.920	0.649
***DGAT2*** **rs3060**							
Smoking	0.024	0.020	0.153	0.009	0.130	0.004	0.342
Drinking	0.265	0.260	<0.001	0.443	0.021	0.512	0.047
***MOGAT2*** **rs600626**							
Smoking	0.620	0.880	0.237	0.516	0.537	0.433	0.928
Drinking	0.353	0.946	0.752	0.393	0.961	0.364	0.522
***MOGAT2*** **rs609379**							
Smoking	0.488	0.280	0.107	0.990	0.190	0.186	0.828
Drinking	0.007	0.236	0.190	0.353	0.269	0.442	0.912
***MOGAT2*** **rs10899104**							
Smoking	0.374	0.115	0.033	0.292	0.115	0.004	0.629
Drinking	0.150	0.505	0.057	0.485	0.073	0.486	0.605

*The abbreviations of SNP, TC, TG, HDL-C, LDL-C, ApoA1 and ApoB respresent single nucleotide polymorphism, total cholesterol, triglyceride, high-density lipoprotein cholesterol, low-density lipoprotein cholesterol, apolipoprotein A1 and apolipoprotein B; respectively. P_I_ < 0.005 was considered statistical significance after the Bonferroni correction (5 SNPs × 2 risk factors)*.

### SNP-Smoking/Drinking Interactions on the Risk of IS

As presented in Table 7, the SNPs did not interact with smoking or drinking to influence the risk of IS (*P*_I_ > 0.005 for all).

**Table 7:** Interactions of the genotypes and smoking /drinking on the risk of IS.

**SNP/factor**	**Genotype**	**OR(95%CI)**	***P*_I_**	**SNP/factor**	**Genotype**	**OR(95%CI)**	***P*_I_**
*DGAT2* rs11236530				*DGAT2* rs11236530			
**Non-smoking**	CC	1.00		**Non-drinking**	CC	1.00	
	CA	1.34 (0.95–1.89)			CA	1.28 (0.92–1.79)	
	AA	2.94 (0.90–9.56)	0.77		AA	1.82 (0.97–3.42)	0.53
**Smoking**	CC	1.00		**Drinking**	CC	1.00	
	CA	1.43 (0.93–2.21)			CA	2.58 (1.00–2.49)	
	AA	1.90 (0.96–3.74)			AA	3.82 (0.96–5.27)	
*DGAT2* rs3060				*DGAT2* rs3060			
**Non-smoking**	TT	1.00		**Non-drinking**	TT	1.00	
	CT	1.04 (0.74–1.48)			CT	1.35 (0.96–1.89)	
	CC	1.34 (0.70–2.54)	0.65		CC	1.75 (1.00–3.08)	0.16
**Smoking**	TT	1.00		**Drinking**	TT	1.00	
	CT	1.20 (0.80–1.83)			CT	0.79 (0.51–1.23)	
	CC	2.17 (0.87–5.43)			CC	1.08 (0.23–5.04)	
*MOGAT2* rs10899104							
**Non-smoking**	TT	1.00					
	TG	1.14 (0.81–1.60)					
	GG	1.89 (0.72–4.97)	0.37				
**Smoking**	TT	1.00					
	TG	1.20 (0.78–1.84)					
	GG	0.86 (0.43–1.70)					

*IS, ischemic stroke; OR, odds ratio; CI, confidence interval; P_I_, the interaction P-value*.

### Haplotype-Smoking/Drinking Interactions on the Risk of IS

The haplotype-smoking interaction on the risk of IS was not detected in the present study. As compared with the commonest haplotype of the rs600626A-rs609379C-rs108991045T in nonsmokers, the haplotype of rs600626G-rs609379A-rs108991045G (OR = 0.55, 95% CI = 0.32–0.97) in smokers decreased the risk of IS, but the difference was not statistically significant (*P*_I_ = 0.067; Table 8).

**Table 8:** Interactions of the haplotypes and smoking on the risk of IS.

**Haplotype**	**Frequency**	**OR (95%CI)_non−smoking_**	**OR (95%CI)_smoking_**
A-C-T	0.6133	1.00	1.03 (0.69–1.52)
G-A-G	0.1142	0.76 (0.50–1.16)	0.55 (0.32–0.97)
G-C-T	0.0792	0.77 (0.53–1.13)	1.73 (0.69–4.34)
A-C-G	0.0785	1.44 (0.83–2.51)	0.83 (0.51–1.35)
*P*_I_ = 0.067			

*IS, ischemic stroke; OR, odds ratio; CI, confidence interval; P_I_, the interaction P-value*.

Several haplotype-alcohol interactions on the risk of IS were observed in the present study (*P*_I_ < 0.0001; *P*_I_ < 0.006 was considered statistical significance after the Bonferroni correction: 4 haplotypes × 2 risk factors). In comparison with the commonest rs600626A-rs609379C-rs10899104T haplotype in nondrinkers, the rs600626G-rs609379C-rs10899104T haplotype in non-drinkers was associated with increased risk of IS (OR = 5.24, 95% CI = 2.64–10.04). However, the rs600626G-rs609379A-rs10899104G (OR = 0.41, 95% CI = 0.22–0.76) and rs600626G-rs609379C-rs10899104T (OR = 0.12, 95% CI = 0.04–0.36) haplotypes in drinkers were associated with decreased risk of IS compared with the commonest rs600626A-rs609379C-rs10899104T haplotype in drinkers (Table 9).

**Table 9:** Interactions of the haplotypes and drinking on the risk of IS.

**Haplotype**	**Frequency**	**OR (95%CI)_non−drinking_**	**OR (95%CI)_drinking_**
A-C-T	0.6133	1.00	0.87 (0.58–1.31)
G-A-G	0.1145	0.81 (0.55–1.21)	0.41 (0.22–0.76)
G-C-T	0.0813	5.24 (2.64–10.04)	0.12 (0.04–0.36)
A-C-G	0.0783	1.32 (0.87–1.99)	0.62 (0.35–1.09)
*P*_I_ < 0.0001			

*IS, ischemic stroke; OR, odds ratio; CI, confidence interval; P_I_, the interaction P-value*.

## Discussion

The major results of the present study are as follows: (1) Both genotype and allele frequencies of the rs11236530 SNP were different between IS and control groups, the rs11236530CA/AA genotypes and A allele frequencies were higher in IS patients than in controls. (2) The rs11236530 SNP was associated with increased risk of IS (CA/AA *vs*. CC, OR = 1.45, 95% CI = 1.12–1.88, *P* = 0.0044). (3) The rs600626G-rs609379A-rs10899104G haplotype decreased the risk of IS (adjusted OR = 0.67, 95% CI = 0.48–0.93, *P* = 0.018). (4) Serum HDL-C concentrations in controls were different among the rs11236530 genotypes, the rs11236530A allele carriers had lower HDL-C levels than the rs11236530A allele non-carriers. (5) The rs11236530, rs3060 and rs10899104 SNPs interacted with cigarette **s**moking to influence serum ApoB levels, whereas the rs11236530 and rs3060 SNPs interacted with alcohol consumption to affect serum HDL-C levels (*P*_I_ < 0.004–0.001). (6) Several haplotype-alcohol consumption interactions on the risk of IS were also observed. The rs600626G-rs609379A-rs10899104G-alcohol (OR = 0.41, 95% CI = 0.22–0.76) and rs600626G-rs609379C-rs10899104T-alcohol (OR = 0.12, 95% CI = 0.04–0.36) interactions decreased the risk of IS.

The genotype and allele frequencies of the *DGAT2-MOGAT2* SNPs in different populations are not well-known. A previuos large meta-analysis of lipid phenotypes with the use of a dense gene-centric approach showed that the rs11236530A allele frequency was 42.46% (28). The frequencies of rs3060TT, TC and CC genotypes in Chinese patients with dyslipidemia were 43.6, 41.0, and 15.4% (45) or 51.1, 40.5, and 8.4% (52); respectively. In the International 1,000 Genomes database (https://www.ncbi.nlm.nih.gov/variation/tools/1000genomes/), the rs11236530A allele frequency was 16.15% in African Carribbean individuals in Barbados (ACB); 13.93% in Americans of African Ancestry in the Southwestern USA (ASW); 9.88% in Bengali from Bangladesh (BEB); 25.27% in Chinese Dai in Xishuangbanna, China (CDX); 10.10% in Utah residents (CEPH) with Northern and Western European Ancestry (CEU); 20.39% in Han Chinese in Beijing, China (CHB); 16.67% in Southern Han Chinese (CHS); 5.85% in Colombians from Medellin, Colombia (CLM); 11.62% in Esan in Nigeria (ESN); 6.57% in Finnish in Finland (FIN); 9.89% in British in England and Scotland (GBR); and 8.25% in Gujarati Indian from Houston, Texas (GIH). The rs3060C, rs600626G, rs609379A, and rs10899104G allele frequencies were also different in the abovementioned ethnic groups. In the Chinese populations, the rs3060C, rs600626G, rs609379A, and rs10899104G allele frequencies in CDX, CHB and CHS were 27.96, 22.30, and 20.48%; 23.66, 22.33, and 20.00%; 19.89, 15.53, and 20.48%; and 24.73, 21.84, and 24.29%; respectively. In the current study, we found that the rs11236530CA/AA genotype and A allele frequencies were higher in IS than in control groups. These findings suggest that some *DGAT2-MOGAT2* SNPs may be different in distinct races, ethnic groups, or populations. We found that the prevalence of the rs11236530A allele was higher in Chinese than in Europeans or African. These results might also be a reasonable explanation for the distinct prevalence of IS between Chinese and European or African.

The association between the *DGAT2-MOGAT2* SNPs and IS remains unknown. In the present study, we showed, for the first time to our knowledge, that the rs11236530 SNP increased the risk of IS in different genetic models. In addition, we also found moderate LD among the rs600626, rs609379 and rs10899104 SNPs in our study populations. The rs600626G-rs609379A-rs10899104G haplotype decreased the risk of IS (adjusted OR = 0.67, 95% CI = 0.48–0.93, *P* = 0.018). Several haplotypes interacted with alcohol consumption to influence the risk of IS. The rs600626G-rs609379A-rs10899104G-alcohol (OR = 0.41, 95% CI = 0.22–0.76) and rs600626G-rs609379C-rs10899104T-alcohol (OR = 0.12, 95% CI = 0.04–0.36) interactions decreased the risk of IS. These results suggest that the rs11236530 SNP may be a new genetic marker for ischemic cardiovascular disease.

The potential association between the *DGAT2-MOGAT2* SNPs and blood lipid concentrations in humans has not been well-elucidated. In a previous large-scale gene-centric meta-analysis across 32 studies, Asselbergs et al. (28) showed that the rs11236530 SNP decreased HDL-C levels in the European populations. Although the rs3060 SNP was not associated with blood lipid traits in previous GWASes (19, 20, 22), the variant allele was significantly associated with liver fat content changes in response to niacin treatment (45). Liver fat content was lower in subjects with two copies of the variant allele than in the homozygous wild-type. The rs3060 SNP was not related to the baseline liver fat content or other parameters, or changes in body weight, visceral adipose tissue, plasma TG, free fatty acid, insulin, or liver enzyme level. In the current study, we revealed that serum HDL-C concentrations in controls were significantly different among the rs11236530 genotypes, the subjects with rs11236530CA/AA genotypes had lower HDL-C concentrations than those with the rs11236530CC genotype. In addition, this study is the first to report that the rs11236530, rs3060 and rs10899104 SNPs interacted with cigarette **s**moking to influence serum ApoB concentrations; and the rs11236530 and rs3060 SNPs interacted with alcohol consumption to affect serum HDL-C concentrations.

This investigation may have several limitations. First, the number of both control and patient groups was relatively small compared with some previous genetic studies. Second, many IS patients were taking some drugs that may affect serum lipid profiles. Therefore, we could not determine the association between the *DGAT2-MOGAT2* SNPs and serum lipid levels in IS group. Third, although several factors were adjusted for the statistical analyses, some clinical characteristics were different between the two groups. Finally, there are still many unmeasured environmental and genetic factors and their interactions in this study. Thus, further large studies are needed to confirm our findings.

## Conclusion

This study shows that the rs11236530A allele frequency was higher in IS patients than in controls. The rs11236530 SNP and rs600626G-rs609379C-rs10899104T haplotype carriers in nondrinker were associated with increased risk of IS, whereas the rs600626G-rs609379A-rs10899104G haplotype; and the rs600626G-rs609379A-rs10899104G-alcohol and rs600626G-rs609379C-rs10899104T-alcohol interactions were associated with decreased risk of IS. The rs11236530A allele carriers in controls had lower HDL-C levels than the rs11236530A allele non-carriers. The rs11236530, rs3060 and rs10899104 SNPs interacted with cigarette smoking to influence serum ApoB levels, and the rs11236530 and rs3060 SNPs interacted with alcohol consumption to affect serum HDL-C levels. These results suggest that the rs11236530 SNP may be a new genetic marker for IS. The association between the rs11236530 SNP and the IS risk may be partly explained by decreasing serum HDL-C levels in our study populations.

## Data Availability Statement

The original contributions presented in the study are included in the article/Supplementary Material, further inquiries can be directed to the corresponding author/s.

## Ethics Statement

The studies involving human participants were reviewed and approved by the Ethics Committee of the First Affiliated Hospital, Guangxi Medical University (No. Lunshen 2014-KY-Guoji-001; Mar. 7, 2014). The patients/participants provided their written informed consent to participate in this study.

## Author Contributions

Y-GZ conceived the study, participated in the design, helped to undertake genotyping, performed the statistical analyses, and drafted the manuscript. R-XY and X-LC conceived the study, participated in the design, collected the clinical data and samples, and helped to draft the manuscript. FH, J-ZW, and W-XC collaborated to the genotyping and collected the clinical data and samples. All authors contributed to the article and approved the submitted version.

## Funding

This study was supported by the National Natural Science Foundation of China (No. 81460169) and the Youthful Science Foundation of Guangxi Province (No. 2017GXNSFBA198067).

## Conflict of Interest

The authors declare that the research was conducted in the absence of any commercial or financial relationships that could be construed as a potential conflict of interest. The reviewer S-LP declared a shared affiliation, with no collaboration, with the authors to the handling editor at the time of the review.

## Publisher's Note

All claims expressed in this article are solely those of the authors and do not necessarily represent those of their affiliated organizations, or those of the publisher, the editors and the reviewers. Any product that may be evaluated in this article, or claim that may be made by its manufacturer, is not guaranteed or endorsed by the publisher.
